# Usability on the p-medicine infrastructure: an extended usability concept

**DOI:** 10.3332/ecancer.2014.399

**Published:** 2014-02-11

**Authors:** Marie-Luise Christ-Neumann, Ana Escrich, Alberto Anguita, Holger Stenzhorn, Marian Taylor, Hena Ramay, Stefan Rüping, Christian Krauth, Wolfgang Kuchinke, Norbert Graf, Simona Rossi

**Affiliations:** 1 Fraunhofer-Gesellschaft zur Förderung der Angewandten Forschung e. V. Institute for Intelligent Analysis and Information Systems (IAIS) Schloss Birlinghoven, 53754 Sankt Augustin, Germany; 2 Universidad Politécnica de Madrid, Facultad de Informática, Campus de Montegancedo, Boadilla del Monte, 28660 Madrid, Spain; 3 Department of Paediatric Oncology and Haematology, Saarland University, Campus Homburg, Building no. 9, 66421 Homburg, Germany; 4 University of Oxford, Wellington Square, Oxford OX1 2JD, UK; 5 SIB | Swiss Institute of Bioinformatics, Quartier Sorge, Bâtiment Génopode, CH-1015 Lausanne, Switzerland; 6 Coordination Centre for Clinical Trials (KKS), Heinrich-Heine-University, Moorenstr. 5, 40225 Düsseldorf, Germany

**Keywords:** health care, evaluation, good clinical practice

## Abstract

Usability testing methods are nowadays integrated into the design and development of health-care software, and the need for usability in health-care information technology (IT) is widely accepted by clinicians and researchers. Usability assessment starts with the identification of specific objectives that need to be tested and continues with the definition of evaluation criteria and monitoring procedures before usability tests are performed to assess the quality of all services and tasks. Such a process is implemented in the p-medicine environment and gives feedback iteratively to all software developers in the project. GCP (good clinical practice) criteria require additional usability testing of the software. For the p-medicine project (www.p-medicine.eu), an extended usability concept (EUC) was developed. The EUC covers topics like ease of use, likeability, and usefulness, usability in trial centres characterised by a mixed care and research environment and by extreme time constraints, confidentiality, use of source documents, standard operating procedures (SOA), and quality control during data handling to ensure that all data are reliable and have been processed correctly in terms of accuracy, completeness, legibility, consistence, and timeliness. Here, we describe the p-medicine EUC, focusing on two of the many key tools: ObTiMA and the Ontology Annotator (OA).

## Background

Usability is the measure of the potential of the software to accomplish the goals of the user including ease of use, visual consistency, and so on (definition by TechTarget, http://searchsoa.techtarget.com/definition/usability). In recent years, usability testing has become of more and more importance in the field of software and interface development. Software should support the user in his/her daily work, especially when various user groups are working with the same platform or tools. This is a great challenge for the European research project p-medicine (http://www.p-medicine.eu), where a service-oriented clinical research infrastructure is under development to improve the prognosis of patients by paving the way to personalised medicine. The developed tools have to be tested by the prospective end-users (clinicians, bioinformaticians, statisticians, data managers, researchers, and patients) and evaluated by a usability engineer throughout the whole developmental period within the p-medicine environment. Furthermore, the usability of p-medicine tools will also be evaluated in an international clinical research infrastructure (ECRIN, European Clinical Research Infrastructures Network, a sustainable, not-for-profit infrastructure supporting multinational clinical research projects in Europe), for employment in international clinical trials. Therefore, compliance with GCP has become part of the usability concept as well as international aspects of clinical trials relevant for investigators [1–3]. GCP is an international ethical and scientific quality standard for designing, recording, and reporting trials that involve the participation of human subjects [4]. It ensures that the use of software tools does not lead to an increased risk for the patient, protects the patient’s rights, and guarantees the ethical conduct of research and the high quality of collected data.

Therefore, our usability concept not only has to cover topics like ease of use, likeability, and usefulness, but also has to be extended to cover conditions of usability in trial centres characterised by a mixed care and research environment and by extreme time constraints, confidentiality, use of source documents, SOA, and quality control during data handling.

The GCP requirements for the area of data management in clinical trials are mostly unspecific at the technical level (e.g., necessity for data privacy, security system, and audit trail). In general, quality control should be applied at each stage of data handling to ensure that all data are reliable and have been processed correctly. The investigator should ensure the accuracy, completeness, legibility, and timeliness of the data reported in the case report forms (CRFs). Data reported on the CRF that are derived from source documents should be consistent with the source: any change or correction to a CRF should be dated, initiated, and explained, and should not obscure the original entry (audit trail).

In p-medicine, various tools and services are under development. Here, we focus on two key tools: the ontology-based clinical trial management application ObTiMA and the OA.

### ObTiMA

ObTiMA (ontology-based trial management application) supports the various user groups to design and manage clinical trials and to collect patient data [5]. Traditionally, this was done using paper-based data collection methods that are time consuming, expensive, and prone to errors.

In recent years, electronic data capture has become widely used for data collection in clinical trials. The pharmaceutical companies and clinical research organisations have developed various methods to make this process user friendly and manageable from the data cleaning angle and flexible enough to be re-usable for different studies. According to ICH-GCP guidelines [6], certain features of data capture practices are requested; therefore, users in this field expect a system to provide these standards.

**Figure 1.**
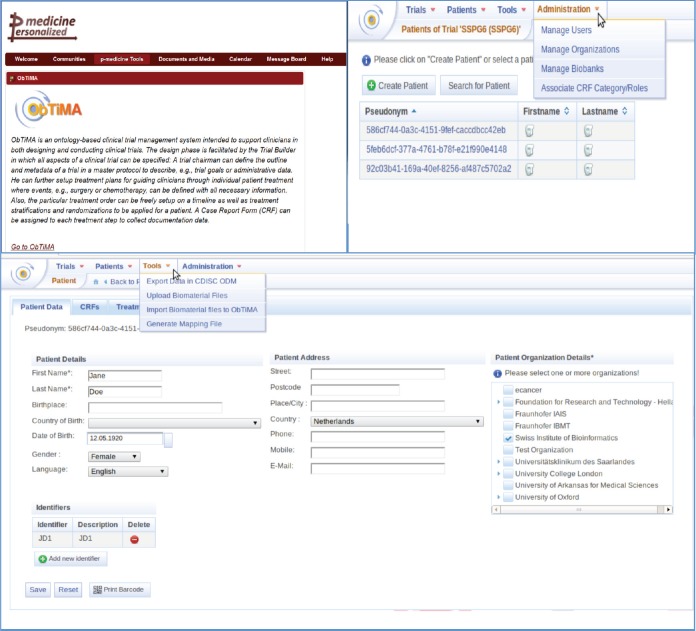
A short description of the functionality of the clinical tool ObTiMA as it can be seen from the portal (upper left corner) and from the test server (upper right corner and bottom panel).

A user has the possibility to design, develop, and conduct clinical trials using ObTiMA. This software goes far beyond a pure data management system. A screenshot is shown to give a first overview of the functionality of ObTiMA and shows the main menu items of the p-medicine platform (Figure 1). ObTiMA is available at: http://obtima.org/. ObTiMA is based on the HDOT (Health Data Ontology Trunk) ontology [7].

The tool includes several canvases for graphically representing both the schema of the database to align, HDOT, and the mappings between views from both models.

### Ontology Annotator

**Figure 2.**
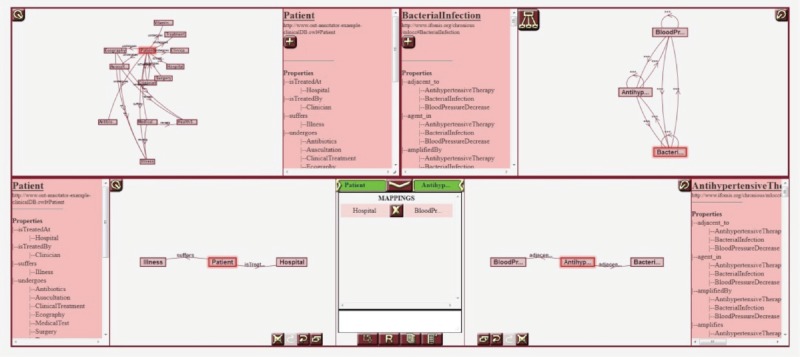
A screenshot of the Ontology Annotator. The tool includes several canvases for graphically representing both the schema of the database to align, HDOT, and the mappings between views from both models.

The p-medicine project incorporates a data integration layer designed to offer researchers a homogeneous view of disparate biomedical data sources—that is, clinical databases, genomic data sources, image repositories, etc. The homogenisation process of the integrated sources is based on a semantic annotation of the source schemas—that is, the alignment of its elements with the HDOT ontology, which acts as a common vocabulary. These semantic annotations are manually defined by end-users. To assist users in the annotation process, the p-medicine platform includes a specific-purpose tool: the OA [8]. The OA is a web-based tool with a graphical interface that allows users to visually navigate through an RDF-based (RDF: resource description framework, http://www.w3.org/RDF/) database schema and through the HDOT ontology and define matches between their elements. Figure 2 shows a screenshot of the OA.

The target users of the OA are generally the owners of the databases themselves who wish to incorporate their data into p-medicine, since the semantic annotation process requires understanding the structure of the database being annotated. In most situations, they will be database administrators rather than clinicians, thus having some degree of expertise in database design and administration. However, the semantic annotation process requires a degree of technical knowledge, which cannot be assumed for every potential user. In the first place, the format of the annotated databases is always RDF—for other formats, for instance Access, Excel, relational, and so on, the OA performs an automatic translation of the database structure into RDF. The RDF paradigm is widely used in biomedical research environments and in integration applications, since it facilitates the formalisation of knowledge domains with little constraints. In the second place, the annotation process itself—especially, when the annotated databases are described in RDF—implies understanding concepts inherent to the alignment of data sources itself. As it cannot be assumed that every user will have expertise in these technologies, great effort has been put in delivering a tool that assists users and hides to a great extent the complexities related to such technologies. Usability has therefore played a central role in the development of the OA. The OA also provides advanced alignment capabilities that differentiate it from former RDF alignment tools, based on the definition of views representing combinations of concepts in the aligned database. This approach enables the translation of data even under the presence of complex semantic heterogeneities. Conducted tests with sample data have already proven the viability of this approach for integrating data from independent clinical trials, enabling researchers to perform knowledge discovery processes on the p-medicine platform.

### Usability testing

In both cases (ObTiMA and OA), the design of the usability testing has been organised in two main phases:

1. Use case-oriented usability with no prior instruction given to the user (internal and external), so that the test is of how intuitive the system is for him/her. This is very worthy to the extent that there might be some issues that are seen as flaws, but in fact could easily have been avoided with the aid of brief instructions. However, in the setting of a clinical unit participating in clinical trials of investigational medicinal products (CTIMPs), trial coordinators are bound by the standards of ICH-GCP to ensure that the trial personnel (including data entry) are eligible to undertake their role in the trial, by education, experience, and training. It is very common for the coordinator to formally plan training in (or at least discussion of) the data capture system, amongst other things (started the first year of the project, 2011, and still ongoing).
2. Usability within the ECRIN infrastructure including evaluation of legal and ethical framework based on international requirements, data security, anonymisation, pseudonymisation, and system validation (will be performed in 2014, validation simulation performed). The evaluation is maturity based (requirements have been set up and tool maturity has been assessed).

## Methods

### Phase1

**Figure 3.**
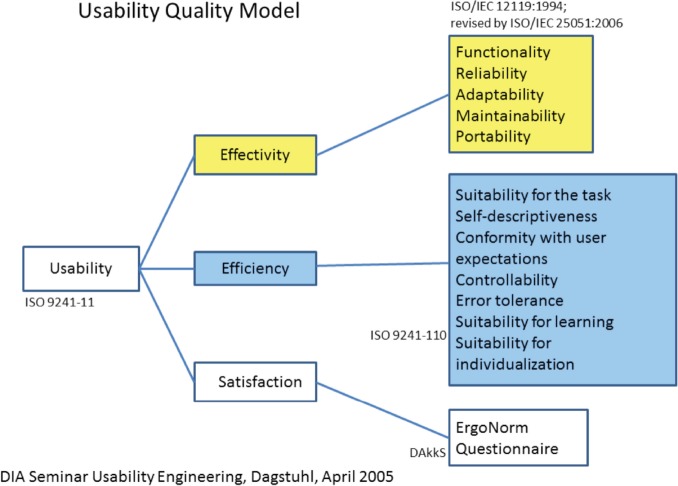
Usability quality model as adopted in phase 1.

**Figure 4.**
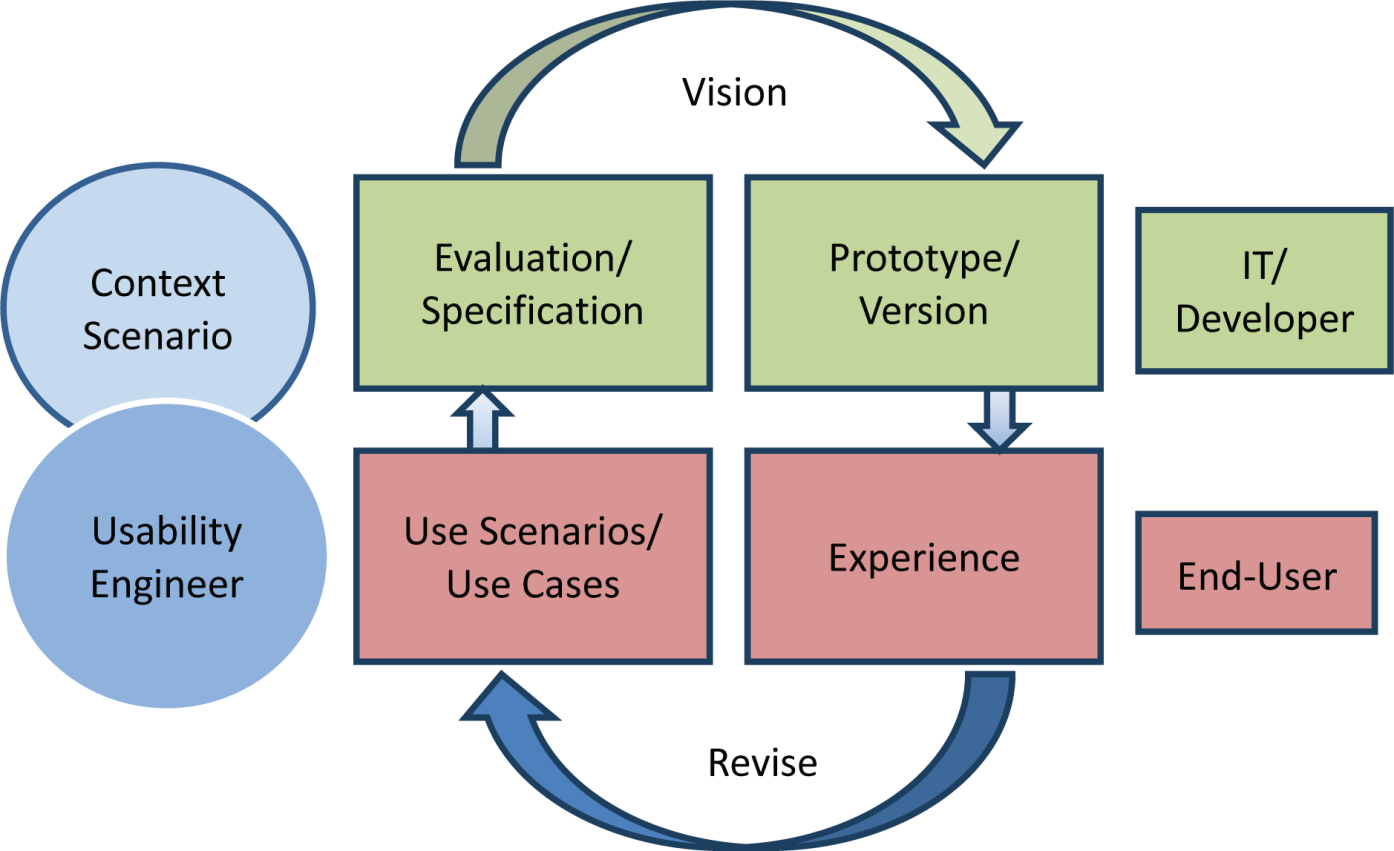
The development Loop highlights the iterative process usability testing and evaluation/modification of the tools.

Many standards are related to or affect the usability of computer software and applications. These standards have to be taken into account during the whole developmental process. For the first phase of usability evaluation regarding developed software and tools, we focused on ISO 9241 [9] and the ISO-based DAkkS usability method [10]. The figure of the usability engineer (UE) is an independent agent between the end-users and the developers, and it describes a pragmatic approach that emphasises empirical methods and operational definitions of user requirements for tools concerning software ergonomic standards. The DAkkS usability method is based on the ergonomics of human system interaction ISO 9241, in particular on part 11 [11] and part 110 [12] (details are shown in Figure 3). It describes the requirements for usability and offers guidance for practitioners to conduct usability tests of interactive software. In part 11, the usability process is described as the ‘extent to which a product can be used by specified users to achieve specified goals with effectiveness, efficiency, and satisfaction in a specified context of use’. This means enabling the end-user to achieve his/her results and to note his/her interests in the relevant context of use recognising the dialogue principles, listed and described in ISO 9241-Part 110. The software has to fit to the users’ needs and not *vice versa*. Usability involves the systematic identification of requirements of the usability process during the whole development loop, shown in Figure 4.

Within p-medicine, the UE identified the user groups and interviewed (documented in the form of context scenarios) several prospective end-users of each user group to understand their tasks and the context of use; the UE elaborated specific key questions in order to retrieve the users’ needs, and finally described the whole context of use: the user’s prior knowledge and qualification, his/her working environment, and his/her specific way of working have been specified. The derived system requirements were in accordance with the seven dialogue principles, described in ISO 9241-110.

Following these principles, the resultant user requirements are not product properties but represent the bridge between problem and concrete solution that enable the user to conduct his/her work with the support of the developed tool efficiently and the software developer to have the understanding of the user’s task to assure a usable user interface that supports the user to achieve his/her aim in an efficient, effective, and satisfied way [13].

Usability tests have been recorded. CamStudio (http://camstudio.org/, free streaming video software) has been a useful tool for the tester as well as for the evaluator. In terms of time, it reduces the tester’s need to document all findings and to illustrate with examples or screen-shots. Thus, a thorough and detailed testing report is provided to the evaluator.

In accordance with the requirement specifications as defined by the context scenarios, the tool implementation was consolidated. With a very early tool prototype, the end-users (internal and external) had the opportunity to test the interface and restricted functionality. This has been an essential step to enable the end-user to work with the developed prototypes since the early stages of development, to demonstrate to the user that the requirements were well understood, to retrieve a first feedback of the interface, and to highlight to what extent the user expectations were met and possibly expand the user requirements.

More advanced prototypes were delivered followed by extensive batteries of usability tests (both internal and external end-users) targeted at delivering sound tools. The conducted tests resulted in an extensive report covering a set of required features and visual enhancement recommendations. Examples of the usage problems that occurred are presented in the Appendix.

### Phase 2

What has been described above is the basic way of evaluating usability during software development, for example by focusing on the quality of interaction between a product (system) and the user [14]. In this case, the unit of measurement is the degree of important human factors, like user satisfaction, comfort, time spent, and so on, in a specific context of use. The distance separating the software designer from the end-user in the interaction mostly depends on different mental views: the designer simulates the interaction or the user interacts with the system. It can be expected that the difference in the mental models is especially pronounced between the software designer and the physician using the software to conduct clinical trials. Because the physician is not only acting as an investigator in a clinical trial but also involved in care activities, time and resource limitations exist. Hence, data collection in the CRF must be fast and without difficulties. The whole process of data collection from defining data sets to data input, checking, and analysing is resource intensive. It utilises sophisticated technologies and employs highly skilled professionals. The results of a clinical trial and the statistical analysis can only be as good as the data collected in the CRF. For this reason, GCP requires, besides the validation of the software solution used, the training of the investigators in using the software.

ECRIN is one of the p-medicine partners who help in answering the need of establishing GCP compliance in p-medicine. ECRIN has developed a standard for the evaluation of data centres that will be employed in p-medicine. Furthermore, ECRIN is creating certified data centres that are able to provide GCP compliant data management. The ‘Working Group on Data Centres’ of ECRIN has developed a standard describing the requirements of GCP-compliant data management in multinational clinical trials [15], which can be used to certify clinical data management centres. These requirements are divided into two main parts: an IT part covering standards for the underlying IT infrastructure and computer systems in general, and a data management (DM) part covering requirements for data management applications in clinical trials. In 2012, the ECRIN standard has been used for two pilot audits of ECRIN data centres and more audits will follow. On the basis of ECRIN’s experience, a new usability phase has been implemented which covers many aspects of the process of usability tests as described in Table 1. Notably, for clinical trials, Ryan et al. [16] described six challenges and solutions if informatics is incorporated into studies. These are

(a) intervention integrity
(b) software updates and compatibility
(c) web accessibility challenges
(d) hardware and equipment challenges
(e) computer literacy of participants
(f) programming challenges.

Importantly, these challenges also cover environmental factors and not only factors directly related to the software. To be successful on the market, usability tests have to cover such challenges as well.

Safety is a further aspect that needs to be taken very seriously in the health-care sector [17, 18]. The American Medical Informatics Association (AMIA) Board of Directors convened a task force on usability to examine evidence from literature and give recommendations to reduce or avoid unintended consequences or harm caused by electronic health records (EHR). They summarised their findings in ten recommendations that are categorised into four areas and listed as follows:

(a) Human factor health IT) research
  1. Prioritise standardised use cases.
  2. Develop a core set of measures for adverse events related to health IT use.
  3. Research and promote best practices for safe implementation of EHR.
  (b) Health IT policy
    1. Standardisation and interoperability across EHR systems should take account of usability concerns.
    2. Establish an adverse event reporting system for health IT and voluntary health IT event reporting.
    3. Develop and disseminate an educational campaign on the safe and effective use of EHR.
    (c) Industry recommendations
      1. Develop a common user interface style guide for select EHR functionalities.
      2. Perform formal usability assessments on patient-safety-sensitive EHR functionalities.
      (d) Recommendations for the clinician end-user of EHR software
        1. Adopt best practices for EHR system implementation and ongoing management.
        2. Monitor how IT systems are used and report IT-related adverse events.

**Figure 5.**
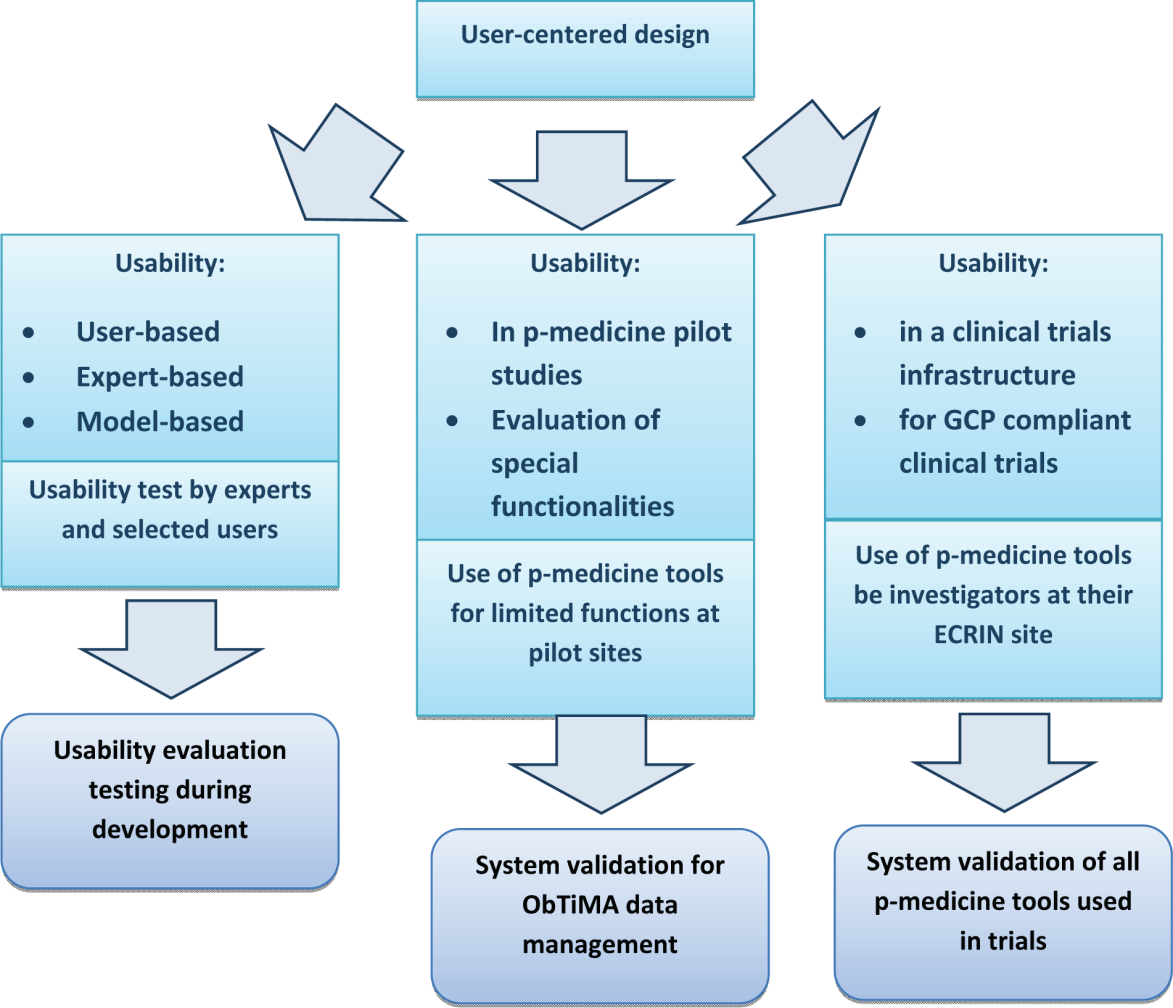
The three columns of usability evaluation and their relation to system validation of p-medicine tools used in clinical trials.

Regulatory authorities expect the management at an investigator site to be in control of all computerised systems, devices, and instruments associated with the delivery of patient care [19]. If data from these systems are used as source data for the CRF, then these data are classified as GCP data. The software that collects and processes patient data and the platform system that allows the software application to work are then considered to be GCP systems and are subject to system validation. In this sense, ObTiMA as well as the OA are GCP systems and should undergo system validation. Figure 5 illustrates the usability evaluation process for tools that involve the use and/or management of clinical trials data.

## Results

### Phase 1

The first feedback of end-users was collected in the first round of usability tests with external volunteers, a clinician, and a study nurse for ObTiMA and a data analyst for the OA. The recorded usability tests are described in a so-called use scenario organised in four columns:

- tasks and sub-tasks;
- actions and comments of the user;
- reaction of the system; and
- list of observed problems involving the dialogue principles with recommendations by the UE.

All unexpected reactions of the system or system failures have been denoted as so-called ‘critical incidents’ which are described in column three and recommended in column four of a use scenario. Important is the ‘thinking aloud’ method of the user during execution of the task, where continuous commentary by the user and reporting of all feelings when working are essential to get feedback on the way the user was working. The user should have a basic comprehension in the use of computers but he/she should not be familiar with the software in order to detect initial usage problems of the software. The problems that occurred were very similar regarding their interaction with the tool, so that we evaluate them together regarding the dialogue principles [12].

#### ObTiMA

The task was to enrol data from a fictional patient into a running trial. Important deficiencies and weaknesses were detected during recording of the interaction of the end-user with the clinical tool. The UE was a participatory observer while the user conducted his/her task. It was a fruitful test for improving the functionality and to guarantee progress.

The process of undertaking data entry also highlighted some potential incompatibilities between the needs of a typical data entry person and the aims of the developer. The general look of the paper form and the screen form was similar. All these issues differentiate a use scenario from a use case, whereby the latter describes only the action of the user and the reaction of the system. Evaluation of these use scenarios and the improvements of ObTiMA in the first prototype influenced the development of the second prototype that was then tested again by internal and external users in the same way. This iterative process takes place during the whole implementation phase and contributes to an ergonomic, qualitative, and usable software tool. Detailed results from the first series of tests are available in Appendix.

#### Ontology Annotator

The task was to map a triple into an HDOT triple (http://www.w3.org/TR/rdf-concepts/#section-triples), and was performed by both internal and external users. The result of the usability process for the OA was (i) a more enhanced tool in terms of efficiency—the added features facilitated user work and reduced the average time needed to create an annotation and (ii) ease of use, a more intuitive interface was achieved. Overall, the performance of usability tests allowed improvement to the quality of the developed tool, providing a more appealing application for end-users. Detailed results are available in Appendix.

Tests’ results with quantity and quality of the testers’ comments allowed the evaluator to discern the successful and well-produced parts of the system as well as to identify situations where it is unclear for the user on how to proceed or the user obtains an unexpected result.

Having the recording and commentaries from a session helped the developers to diagnose problems, since the exact keystrokes that led to the problems were available for review. In addition to undertaking a prearranged set of keystrokes for a set of tasks, it was useful for a tester, experienced in the field to which the development is applied, to ‘play with’ the system.

It has been useful to have several different persons working on the same platform. Collaborating developers, who produce their components independently and then merge them on the main server, and end-users have been working on the system from various bases. Thus, there has also been the opportunity to check whether the system is compatible with a variety of combination of operating systems and browsers.

### Phase 2

Usability and generally quality assurance have been conceived since the beginning of the project as a clear self-improving sequence of in-depth testing of the p-medicine components. In this view, several ad-hoc tasks and interdependent deliverables have been planned to provide sufficient documentation and evidence to evaluate the p-medicine tools within the ECRIN infrastructure. Thus, ECRIN together with other p-medicine members started its process from the description of user needs and requirements. On the basis of these efforts, which have been reported during the project’s lifecycle, scenarios, use cases, and quality reports have been defined to establish requirements for the usability in the ECRIN infrastructure, which in other words means to establish GCP compliance in p-medicine. P-medicine met the requirements for security, legal and ethical framework, that are the base for a correct data use in an international framework. Developers were interviewed to evaluate and establish the tool maturity status; the questionnaire was divided into five sections: software development process, continuous delivery, specification, testing, and quality management and support documents. Each section was then divided into more specific questions that were evaluated as 1 if the task was completed, 0.5 if in progress, and 0 if not yet available. Partial scores were summed and resulted in a score for every section. A comprehensive score led to the association between every tool and a specific maturity level. Score per section was also clustered allowing an immediate visualisation of what tool reached higher maturity and those where more work needs to be performed. The highest score was assigned to ObTiMA that positively answered to almost every question.

## Discussion

The reliability of a computerised system used for clinical trials must be ensured; it rests upon three factors: the quality built into the system during its development and accompanying testing done by the developer, the installation of the system at the investigator site, and the configuration management during the system’s operational use. The usability of the system at the investigator site is of prime importance for GCP. Therefore, it must be determined for what purposes the site uses the newly installed software applications, which of these purposes are in need of GCP compliance, and if a mix of GCP and non-GCP purposes is intended. Usability is assured by appointing, training, and organising an adequate number of competent staff to use the systems according to GCP standards, including the provision of SOPs, instruction sheets, on-line help, and support hotlines. Nonetheless, the users’ compliance with the proper procedures for the GCP system has to be checked. For the evaluation of ObTiMA and OA as part of the clinical trials data management infrastructure within p-medicine, the scenario-based usability followed by the ECRIN standard application has been adopted by p-medicine.

The use of the ECRIN standard for the evaluation of p-medicine has several advantages for p-medicine:

- Use of a well-tried standard, already used in audits.
- It considers a large spectrum of regulations, guidelines, and best practices.
- It can guarantee a standard quality level for data management, based on agreed, minimal requirements for GCP compliance.
- It is an open standard, focused not on the pharmaceutical industry, but on academic centres.
- It can complement different existing approaches and systems.
- Because it consists of an IT and a DM part, it can be employed in a flexible way.
- It is user friendly (a detailed guidance document is provided with the standard).
- It specifically focuses on European clinical trials.
- Its requirements are realistic for academic units.
- Certification according to the standard can be a preparatory step for a full system validation.
- It can be used for training and for informing developers about compliance requirements.

In parallel, p-medicine’s team planned the assessment of the so-called non-functional requirements: scalability and system performance. Performance is characterised by the amount of useful work accomplished by a system compared to the time and resources used; therefore, we will measure among others system responsiveness, resource availability, time needed for time compression and decompression, and data transmission time.

Scalability, intended as the ability of a system to handle a growing amount of work in a capable manner or its ability to be enlarged to accommodate that growth, has to measure the system performance when the number of users, number of datasets, size of datasets, and in general the computational requirements increase. For this purpose, possible causes of bottleneck have been identified in the p-medicine platform and they will be tested in terms of response time, throughput, and use of resources.

## Conclusions

For the usability evaluation of p-medicine tools, an EUC was developed in the p-medicine project consisting of two approaches. First, usability evaluation testing during software development, including usability tests by experts and selected users, and second, evaluation of usability in a clinical trials infrastructure and employment of p-medicine tools for GCP-compliant clinical trials. Evaluation of usability will also be assessed in a number of pilot trials outside of the p-medicine framework, resulting in system validation.

The p-medicine pilot studies play an important part in the evaluation of the developed products for their use in GCP clinical trials: a Wilms tumor trial, four trials with breast cancer, and one trial with acute lymphoblastic leukaemia. Trials are not integral parts of p-medicine, and hence they will be executed independently. On the other hand, they will be used to provide data for input into VPH (Virtual Physiological Human) models and for the evaluation and validation of the developed software within the p-medicine environment. The SIOP Wilms tumor trial will be an international randomised prospective multicentre GCP trial that will serve as a clinical trial to employ the newly developed tools of p-medicine; especially, ObTiMA will be used as a clinical data management system (CDMS). In addition, ObTiMA as a CDMS plays a role in breast cancer phase II pharmacodynamics trials.

The evaluation of usability of p-medicine tools as part of ECRIN will depend on the maturity of the p-medicine tools at the time of evaluation. Not all tools will be in the stage of release candidate or release. Thus, a flexible approach for the evaluation of tools was necessary. ObTiMA met the requirements to be a release candidate and will therefore undergo a validation step consisting of system validation, test trial vs. user requirements, IQ (installation qualification (instrument has been delivered and installed in accordance with manufacturer’s requirements)), OQ (operational qualification (instrument is functioning in accordance with its specification)), PQ (performance qualification (instrument is continuing to meet its specification)), and developer evaluation. For less developed tools (software still in the stages of beta or alpha), like the OA, a ‘light’ version of evaluation, consisting for example of gap analysis, basic functional testing, and interface evaluation, will be sufficient.

## Conflicts of interest

The authors have declared that no competing interests exist.

## Authors’ contributions

All the authors wrote the manuscript and agreed on the final version.

## Figures and Tables

**Table 1.** Aspects that need to be covered by usability tests.

Usability tests’ aspects
• Formulate evaluation criteria, verification procedures, and feedback report guidelines
• Coordinate validation activities
• Evaluate the developed software tools by testing functionalities, accessibility, respect of user needs, data integration, and execution times
• Verification of GCP (good clinical practice):
✓ protection of human rights as a subject in clinical trial
✓ standards on how clinical trials should be conducted
✓ clinical audit: performance will be regularly reviewed to ensure scheduled activities will be properly executed

## Appendix

This iterative usability evaluation process took and still takes place during the whole implementation phase and contributes to an ergonomic, qualitative, and usable software tool [1].

Although limited by the little functionality available, the first series of tests was very fruitful for both sides: the users and the developers. The users have shown the developers how they are working and what they expect when executing their task in an efficient, effective, and satisfied way. All detected problems have then been solved and helped in improving the newer prototypes.

### ObTiMA: encountered usage problems

From the use scenarios, various usage problems, that volunteers of different user groups encountered when conducting their task (enrolling patient data) into a running trial in ObTiMA, can be identified and described.

First, we list the usage problems the clinician had with ObTiMA:

**Table:**

Issue (usage problem)	Answer from the designer
Mandatory fields should be better marked	Improved in the new version
Highlighting or emphasizing information	Improved in the new version
Better contrast of background to foreground colour	Enhanced in the new version
Missing legends	Inserted in the new version
Usual colours for green, red, etc. user has another understanding	Can be solved by the use of a legend
Missing reaction from the system, only refresh of the system is not the expected reaction	Improved system response in the new version
Reaction of the system not visible for the user— doing action once more resulting in double output	Improved system response in the new version
Missing action guided information—unable to enter input for surgery	Messages have been added on the interface in order to guide the user
Double clicking on a button → mistake by the system; only one click is possible—user has to login once more, not efficient	Bug fixed
Changes after save are not intuitive for the user, user need explanation	Improved communication in the new version

Similar usage problems were encountered by the study nurse:

**Table:**

Issue (usage problem)	Answer from the designer
In which format to enter date of birth should be clear for the user	Added hints in the interface
Error message should help the user for clarification	Improved communication in the new version
Reactions of the system are not clear for the user	Improved communication in the new version
Language should be consistent, German or English, also the mouse documentation	For German users, all should be in German,
For the save action, the user should get a response from the system	Improved system response in the new version
For controlling the entered data a print button is essential	In the new version, the user can review the entered data
Coloured dots should be explained in a legend	Added legend
Double clicking on a button → mistake by the system	Fixed
Delete one incorrect input is impossible, should be explained	Fixed
Not completely satisfied because she could not click on surgery for entering data	Bug fixed

### Ontology Annotator: encountered usage problems

Several external users tested the Ontology Annotator. In the following table, we report the issues encountered by one of them during the test:

**Table:**

Issue (Usage Problem)	Answer from the designer
Portal screen uses too much space for the tool description	This has actually to do with the portal implementation and the way they access the OA tool. Discussions between the OA development team and the p-medicine portal developers will take place in order to optimize the visualization area of the OA, removing unnecessary parts (e.g., moving the OA description to a separate window)
HDOT module pop-up window is very unusable	Agreement to work on improving the HDOT module selection window was reached. Yes, I agree this must be improved. In the first place, the displaying of the available modules will be more compact and descriptive, and in alphabetical order. In the second place, a confirmation message that informs the user which modules have been activated will be included
Tool is unusable with small resolutions. This provokes several problems, as the hiding of classes in the HDOT window, of the need for scrolling from left to right when searching for a suitable relation in a view window	Make smaller buttons
	Discuss about the possibility of providing two tool modes: one for large screens and one for small screens that reduces the font size and images
	Point out the ability to enlarge windows in the tutorial
Search results are hard to review	Results will be displayed in alphabetical order, and HDOT element searches will be dynamic so the user gets better feedback of his actions
Non-standard button images	Replace button images with more standard icons

HDOT: Health Data Ontology Trunk.

OA: Ontology Annotator.
